# Mass Effect of Large Pelvic Lipoma Resulting in Femoral Hernia

**DOI:** 10.7759/cureus.61148

**Published:** 2024-05-27

**Authors:** Siobhan K Clifford, Ahmed M Gadoura, Omotolani O Ishola, Masoud Bashir

**Affiliations:** 1 General Surgery, Our Lady of Lourdes Hospital, Drogheda, Drogheda, IRL

**Keywords:** open repair of femoral hernia, hernia repair, pelvic lipomatosis, incarcerated femoral hernia, : emergency general surgery

## Abstract

A 73-year-old lady presented with a three-day history of constipation, vomiting, and abdominal pain. On examination, a right femoral hernia was identified, and this was confirmed on computed tomography imaging with evidence of mechanical small bowel obstruction. There was an incidental finding of a large pelvic lipoma causing a mass effect. This lady underwent open repair of the femoral hernia. Intra-abdominal lipomatosis is a rare finding and can present itself in a variety of manifestations, or it can be identified as an incidental finding on cross-sectional imaging. Bowel obstructions, abdominal pain, lipoma, and abdominopelvic hernias are some examples of symptomatic presentations of intra-abdominal lipomas.

## Introduction

A lipoma can be defined as one of the most common mesenchymal tumors found in the body, composed of adipose cells frequently surrounded by a thin layer of fibrous tissue. They may arise from any location of the body where adipose tissue is typically present, usually within the subcutaneous tissues of the neck, back, abdomen, or thighs [1]. The exact incidence and prevalence of intra-abdominal lipomatosis are unclear, but upon review of the literature, it seems there are more than 50 case reports published to date [2]. In some cases, there is an underlying disorder such as proteus syndrome, benign symmetrical lipomatosis (aka Madelung’s disease), or macrodystophia lipomatosa associated with these presentations; however, many are asymptomatic and represent incidental findings on cross-sectional imaging [3-5].

## Case presentation

A 73-year-old female presented to the emergency department with a three-day history of constipation associated with a two-day history of abdominal pain, vomiting, and an inability to tolerate oral intake. She had no significant medical or surgical history. The pain was located in the right lower quadrant, was constant, non-radiating, and described as colicky. A physical examination yielded a distended and tender abdomen. More notably, a palpable and irreducible right femoral hernia measuring 3x3 cm was located. 

**Figure 1:**
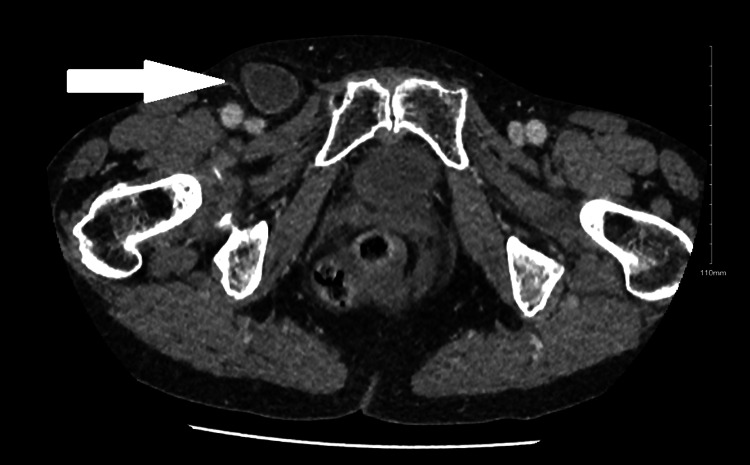
Axial view of right femoral hernia on computed tomography of the abdomen and pelvis.

**Figure 2:**
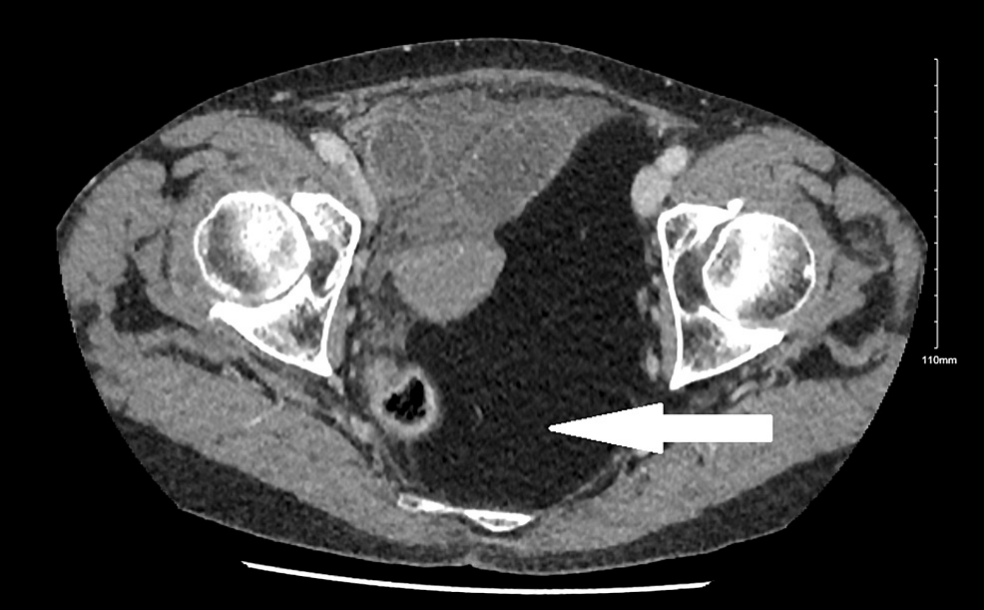
Axial view left-posterior intra-pelvic lipoma on computed tomography abdomen and pelvis.

**Figure 3:**
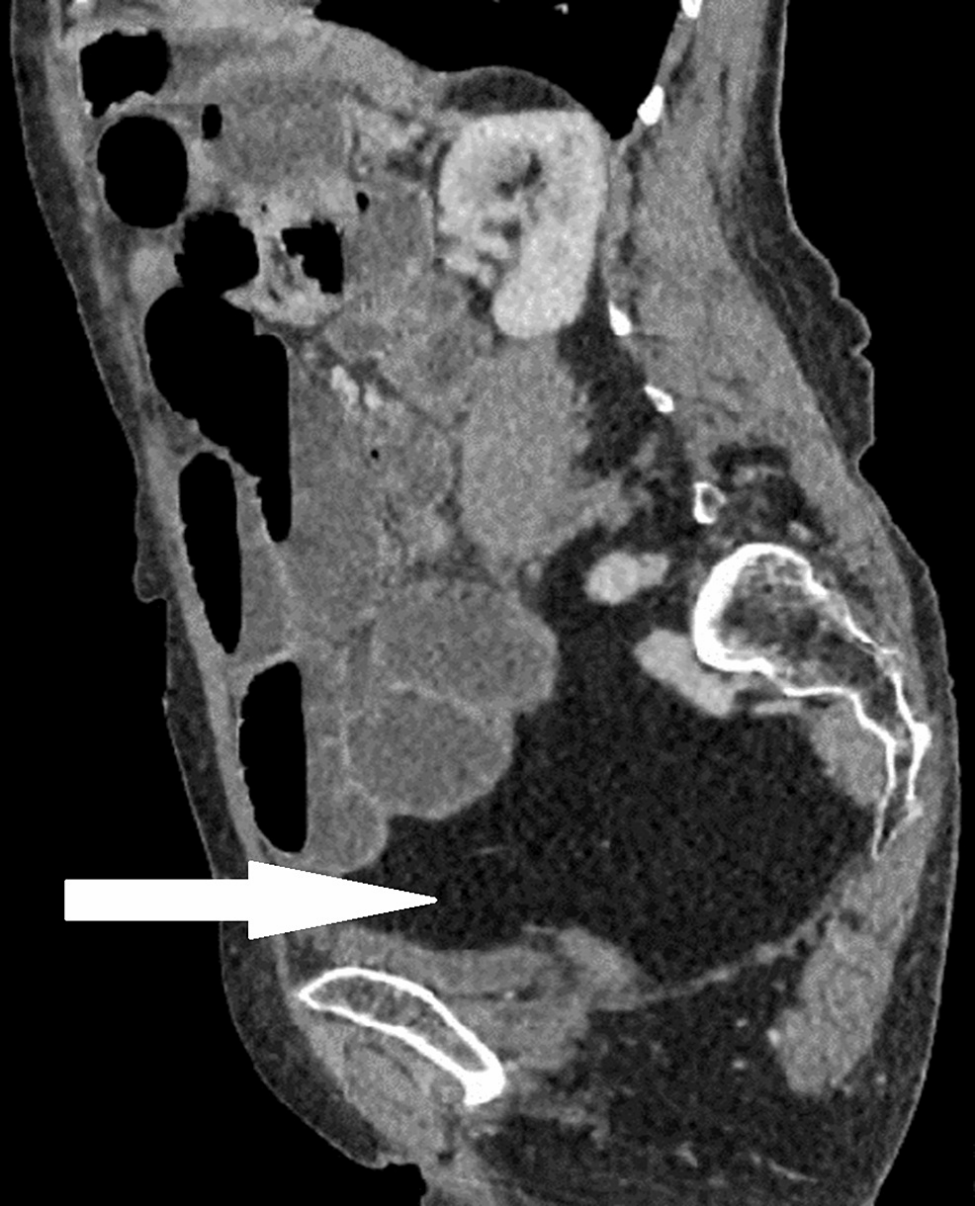
Axial view of left-posterior intra-pelvic lipoma on computed tomography of the abdomen and pelvis.

A diagnosis of small bowel obstruction secondary to a femoral hernia was formulated, indicated by plain film abdomen, and confirmed by computed tomography abdomen and pelvis (CTAP) with contrast. The computed tomography imaging of the abdomen and pelvis (Figures 1-3) revealed evidence of mechanical small bowel obstruction, with the transitional zone located at the level of the strangulated right femoral hernia. Incidentally, a large lipoma on the left side of the pelvis was observed, creating a mass effect and shifting abdominal viscera toward the right side of the pelvis.

An emergency right femoral hernia repair was performed under general anesthesia, which revealed findings of an incarcerated femoral hernia. Small bowel reperfusion was accomplished with the application of warm, moist swabs and 100% high-flow oxygen; no resection was required. The McVey repair was performed, and the patient recovered well in the post-operative period. 

**Figure 4:**
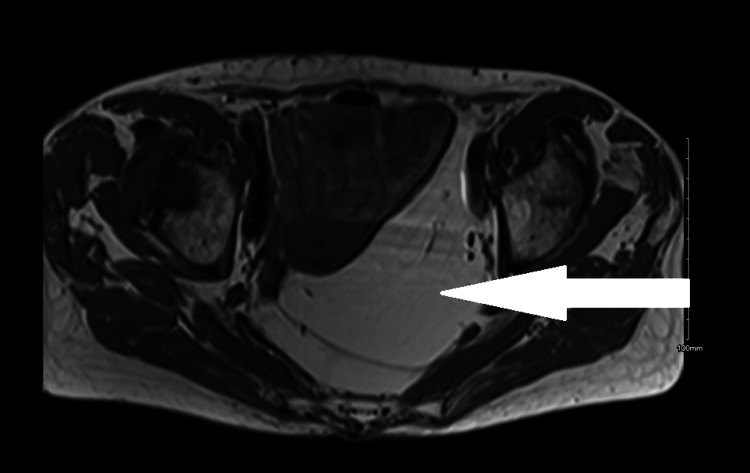
Axial view of pelvic lipoma on magnetic resonance imaging.

Magnetic resonance imaging of the pelvis (Figure 4) was performed to further evaluate the lesion, which illustrated pelvic lipomatosis measuring 9.4 x 11 x 14 cm, predominantly within the posterior and left sides of the hemipelvis, with displacement of pelvic organs and large and small bowels anteriorly and to the right. There were no suspicious features identified. The patient was referred to a specialist center for further evaluation; however, the patient refused a biopsy of the lesion. Further interval imaging revealed an unchanged lipomatosis lesion. 

## Discussion

There are a variety of cases reported in the literature with regard to intra-abdominal lipomas. One such category is that of severe abdominal pain related to torsion of lipomas related to the sigmoid colon and torsion of a pedunculated lipoma arising from the abdominal wall overlying the right iliac fossa, mimicking acute appendicitis [6]. Another clinical presentation associated with these large intra-abdominal lipomas was symptoms of intestinal obstruction [7,8]. 

There are a limited number of case reports in the literature relating to hernias caused by intra-abdominal lipomatosis. Three of those described include a case of a hernia sac containing the uterus associated with a large intra-abdominal lipoma, a sliding indirect lipomatous hernia without a hernial sac, and a sciatic hernia composed of a lipomatous mass transversing the greater sciatic foramen [9-11]. 

Femoral hernias are less commonly identified in the general population and equate to fewer than 4% of all hernias, and there was no case described in the literature pertaining to a femoral hernia in the context of a large intra-abdominal lipoma [12]. The literature reports that 16-23% of emergency scans reveal incidental findings [13,14]. In this instance, the detection of an incidental finding gave greater insight into the etiology of the presentation and facilitated an appropriate follow-up investigation. 

## Conclusions

This case depicts a unique presentation of intra-abdominal lipoma exerting a pressure effect on the viscera and leading to the development of an incarcerated femoral hernia. It illustrates the benefits of pre-operative imaging regarding the detection of incidental findings relating to the presentation. Imaging, in this instance, led to a thorough investigation and the onward referral of the patient to the correct specialist services. In conclusion, the presence of a large pelvic lipomatous lesion leading to an incarcerated femoral hernia is an unusual presentation, and pre-operative imaging allowed for a suitable tertiary referral.
